# Editorial: Neurodevelopment: parental influences, *in utero* exposures, and genetics

**DOI:** 10.3389/fnins.2023.1331453

**Published:** 2024-01-05

**Authors:** Saulo Gantes Tractenberg, Sarah Marie Reinhard, Aaron Sathyanesan

**Affiliations:** ^1^Pontifical Catholic University of Rio Grande do Sul, Porto Alegre, Brazil; ^2^Department of Psychology, Human Arts and Social Sciences, Cabrillo College, Aptos, CA, United States; ^3^Department of Biology, College of Arts and Science, University of Dayton, Dayton, OH, United States; ^4^Department of Electrical and Computer Engineering, School of Engineering, University of Dayton, Dayton, OH, United States

**Keywords:** neurodevelopmental disorders, brain development, attention deficit hyperactivity disorder, maternal immune activation, addiction, SARS CoV-2, microbiome, fetal development

The development of the central nervous system is subject to a broad range of disruptions both environmental and genetic in nature. These disruptions to neurodevelopmental trajectories can result in significant changes to brain structure, connections, and eventually alterations of behavioral outcomes in the short and long-term. Neurodevelopmental disorders and conditions remain a global concern, however, emerging reports indicate that low and middle-income countries bear a more heavy burden related to these conditions (Bitta et al., 2018). While rates of prevalence of neurodevelopmental disorders and related conditions vary between countries, it is reasonable to assume that current estimates are undercounting actual global prevalence of these conditions based on the most updated clinical criteria for diagnoses (Francés et al., 2022). Importantly, pharmacological and other therapeutic interventions have not kept a pace of neurodevelopmental conditions, likely due to a relative lack of basic biomedical research into these conditions.

Although much work remains to be done in the field of neurodevelopmental disorders, over the last half-century there is a greater appreciation of the multifactorial etiology of these conditions. While genetics has long been understood as a critical determinant of these conditions, the developmental environment has received greater scientific and research scrutiny. Importantly, the role of both the prenatal and postnatal environment on cellular and molecular mechanisms affecting neurodevelopment has become a topic of surging interest. This includes important research interfaces with neurodevelopment including the role of the gut-brain axis, substance abuse, viral infections, and signal transduction pathway analysis for diagnostics and therapeutics.

This Research Topic includes nine exciting manuscripts covering important questions across the field of neurodevelopment and the disorders that can result due to specific environmental and genetic disruptions to brain development.

In their impactful research study, Perez et al. tackle the problem of maternal alcohol consumption and its effect on offspring. While a number of studies have investigated the effect of maternal alcohol consumption during the prenatal period, only a handful if any have attempted to define the potential role of maternal alcohol consumption during the lactational period in causing developmental brain deficits in offspring. Using a combination of behavioral, anatomical and cellular-morphological quantification in a novel lactational ethanol exposure mouse model, the authors identify the effects of maternal alcohol consumption during this key postnatal developmental period.

Along a related theme, Crawford et al. in their research manuscript address the important and challenging issue of fentanyl use during pregnancy and neonatal development. Using a rat model of fentanyl use, the authors assess potential effects of this exposure using a battery of behavioral tests at late adolescence. This study lays the groundwork for future preclinical studies on the lasting consequences of fentanyl on neurodevelopment.

Rodent models of neurodevelopmental disorders with complex etiologies often capture different aspects of symptomology; hence it is important to assess potential differences between models using standardized behavioral tests. Carbajal et al. in their research manuscript define the amount of impulsivity in two rat models of attention deficit hyperactivity disorder (ADHD) including spontaneously hypertensive rats and a transgenic rat model in which the ADHD-linked gene Lphn3 has been knocked out. The authors use the delay-discounting task to quantify impulsive choice in both mouse models with interesting differences between the models.

Increasingly, the recognition that neurodevelopmental trajectories can be significantly altered due to infection, neuroimmune responses, and inflammatory processes has come to the fore. Three provocative and insightful review manuscripts cover different angles of the intersection between neurodevelopmental disorders and infectious disease. In their review, Hall et al. take an epidemiological view of neurodevelopmental disorders with an emphasis on maternal immune activation—maternal immune responses that somehow disrupt fetal neural development. Helpfully, the authors also survey important factors that are involved in the onset of neurodevelopmental disorders and how translatable these factors are from animal models to human disorders.

In their review, Recaioglu and Kolk focus on viral infections and how these could potentially alter neurodevelopment. The authors cover more recent viral infections affecting many countries particularly many in the global south including Zika, Chikungunya, and SARS CoV-2. The authors pay special attention to potential routes of entry of these viruses to the developing fetal brain and the cellular populations that are particularly vulnerable to virus-mediated damage.

Zooming in on SARS-CoV-2, Dubey et al. perform a deep dive on the potential ways by which this pandemic-causing virus could affect the prenatal brain, especially considering that early studies indicated that pregnant women were especially vulnerable to COVID-19. The authors cover a lot of ground, looking at clinical evidence from published reports and point to a broad range of cellular, genetic, epigenetic, and brain pathways that future biomedical studies could follow-up on. These studies could then shed light on a disease for which the potential to disrupt neurodevelopmental trajectories appears to be very significant.

The interaction between the maternal microbiome and the developing fetus has been increasingly implicated in affecting long-term neurodevelopmental outcomes of offspring via the maternal-gut-fetal-brain axis. In their important study, Castillo-Ruiz et al. determine if the effects of germ-free gestation—fetal brain development in a maternal environment that is devoid of microorganisms—are more due to *in utero* cellular events or postnatal programming. The authors perform a careful analysis using cellular-morphological techniques as well as gene sequencing analysis to answer this question.

Finally, addressing the molecular and mechanistic bases of neurodevelopmental disorders and conditions we have two review manuscripts covering different signal transduction pathways, both of which have important insights in their particular biological contexts. Ca^2+^ is a critical second messenger in the cell that regulates a host of different cellular processes. Klocke et al. in their manuscript shine a spotlight on available evidence linking Ca^2+^ activity and homeostasis to neurodevelopmental disorders including autism spectrum disorder, ADHD, and schizophrenia.

Neonatal intensive care units (NICUs) have grown significantly efficient over the past 25 years, so much so that neonatal mortality rates have drastically reduced (Driscoll and Ely, 2020). However, certain perinatal complications still cause significant neonatal morbidity. A major cause of neonatal morbidity across the globe is Hypoxic-ischemic encephalopathy (HIE), however, few effective treatment options have been put forward for neonates affected by HIE. Christidis et al. perform a systematic review on a particular class of treatment option offered to HIE-affected neonates—drugs that target the Src kinase signaling pathway. The authors identify evidence on targeting this pathway in preclinical animal models with the hope that this would clarify how effective exactly it is to target the Src kinase pathway in HIE.

In summary, the collection of manuscripts we have edited in this Research Topic represents cutting-edge research papers and reviews across the field of neurodevelopmental disorders and conditions. It is our hope that this Research Topic would help answer important research questions and lead to many more important questions in this field.

## Author contributions

ST: Writing—review & editing. SR: Writing—review & editing. AS: Writing—original draft, Writing—review & editing.

## References

[B1] Bitta M. Kariuki S. Abubakar A. Newton C. (2018). Burden of neurodevelopmental disorders in low and middle-income countries: a systematic review and meta-analysis [version 3; peer review: 2 approved, 2 approved with reservations]. Wellcome Open Res. 2, 121. 10.12688/wellcomeopenres.13540.3 29881784 PMC5964629

[B2] Driscoll A. K. Ely D. M. (2020). Effects of changes in maternal age distribution and maternal age-specific infant mortality rates on infant mortality trends: United States, 2000-2017. Natl. Vital. Stat. Rep. 69, 1–18. Available online at: https://stacks.cdc.gov/view/cdc/89876 32600516

[B3] Francés L. Quintero J. Fernández A. Ruiz A. Caules J. Fillon G.. (2022). Current state of knowledge on the prevalence of neurodevelopmental disorders in childhood according to the DSM-5: a systematic review in accordance with the PRISMA criteria. Child Adolesc. Psychiatry Ment. Health 16, 27. 10.1186/s13034-022-00462-1 35361232 PMC8973738

